# The impact of diabetes on coronary heart disease differs from that on ischaemic stroke with regard to the gender

**DOI:** 10.1186/1475-2840-8-17

**Published:** 2009-03-24

**Authors:** Marjukka Hyvärinen, Jaakko Tuomilehto, Tiina Laatikainen, Stefan Söderberg, Mats Eliasson, Peter Nilsson, Qing Qiao

**Affiliations:** 1 Department of Public Health, University of Helsinki, Helsinki, Finland; 2 Diabetes Prevention Unit, Department of Chronic Disease Prevention, National Institute for Health and Welfare, Helsinki, Finland; 3 Department of Public Health and Clinical Medicine, Umeå University Hospital SE-901 85 Umeå, Sweden; 4 Department of Clinical Sciences, Lund University University Hospital, S-205 02 Malmö, Sweden

## Abstract

**Background:**

To study the diabetes related CVD risk between men and women of different ages.

**Methods:**

Hazards ratios (HRs) (95%CI) for acute CHD and ischaemic stroke events were estimated based on data of Finnish and Swedish cohorts of 5111 women and 4167 men.

**Results:**

182 (3.6%) women and 348 (8.4%) men had CHD and 129 (2.5%) women and 137 (3.3%) men ischaemic stroke events. The multivariate adjusted HRs for acute CHD at age groups of 40–49, 50–59 and 60–69 years were 1.00 (1.94), 1.78 (4.23), 3.75 (8.40) in women (men) without diabetes and 4.35 (5.40), 5.49 (9.54) and 8.84 (13.76) in women (men) with diabetes. The corresponding HRs for ischaemic stroke were 1.00 (1.26), 2.48 (2.83) and 5.17 (5.11) in women (men) without diabetes and 4.14 (4.91), 3.32 (6.75) and 13.91 (18.06) in women (men) with diabetes, respectively.

**Conclusion:**

CHD risk was higher in men than in women but difference reduced in diabetic population. Diabetes, however, increased stroke risk more in men than in women.

## Background

Coronary heart disease (CHD) and stroke are common causes of disability and death [1]. Diabetes mellitus has been found to increase the risk of CHD and stroke events both in women and in men [2-8]. A more markedly increased relative risk for CHD [2,4,5] and stroke [6-8] has been found in diabetic women compared with diabetic men, however the reported magnitudes vary greatly between studies [2,7,8].

CHD and stroke are generally discussed as two diseases with similar etiology caused by atherosclerotic vascular disease. While CHD and stroke have been shown to share some known cardiovascular disease (CVD) risk factors [1], some common CHD risk factors, such as serum total cholesterol [9,10] levels have not been indicated to have an association with increased risk of stroke [10,11]. Also, MONICA study reported CHD rates to decrease and stroke rates to increase in some populations while in other populations the opposite was observed [12]. This may imply different underlying mechanisms and etiology for the two diseases. To what extent diabetes, gender and age affect the development of CHD and stroke is not fully elucidated. Furthermore, little is known about the magnitude of the association between CVD risk factors and the risk of CHD and ischaemic stroke.

The purpose of the present study was to estimate the impact of diabetes and gender and age on the development of CHD and ischaemic stroke and to find out to what extent the known CVD risk factors affect the development of these diseases.

## Participants and methods

This sub-data analysis of the DECODE study comprised of 9278 participants, 5111 (55.1%) women and 4167 (44.9%) men, from 7 cohorts from Finland and Sweden. Individuals participating at the baseline survey were followed up till 2006 and 2004 for the Finnish and the Swedish cohorts respectively. The age range of the study population varied from 40 to 69 years. Measurements for fasting plasma glucose and 75 g 2-h oral glucose tolerance test, body mass index (BMI), total serum cholesterol, HDL cholesterol, systolic and diastolic blood pressure, antihypertensive treatment, and smoking status were available for each study population included in the current analysis.

### Definition of the clinical end points

Acute CHD and ischaemic stroke events (both fatal and non-fatal) were used as end points. Individuals with a history of CVD before the baseline survey were excluded from the analysis. The information on CVD mortality and morbidity were collected from National Causes of Death Register and the National Hospital Discharge Registry and ascertained by using a computerized record linkage of individual ID numbers of each of the individuals participating in the study. The acute CHD and ischaemic stroke events were coded according to the International Classification of Diseases (8^th^, 9^th ^revisions and 10^th ^revision) with codes 410 to 414 and I20 to I25 for fatal and codes 410 to 411 and I21 to I22 for non-fatal CHD and codes 433, 434 and I63 for ischaemic stroke. Cases of haemorrhagic stroke were checked and not included in the final data analysis due to the small numbers. Diabetes was defined as either a history of diabetes at baseline or FPG levels >= 7.0 mmol/L and/or 2-h PG levels >= 11.1 mmol/L. Participants who had emigrated and whose vital status could not be confirmed were treated as censored cases.

The local Ethics Committees had approved each individual study plan and the data analysis plan was approved by the Ethics Committee of the National Public Health Institute, Finland.

### Statistical methods

The data analysis was carried out by using SPSS for Windows version 15.0. Event rates per 1000 person-years for CHD and ischaemic stroke were calculated for per 10-year age-groups (40–69 years), gender and diabetic status. Differences between women and men within each of the age categories were estimated using the χ^2 ^test. Univariate analysis of variance was used to estimate the trend in cardiovascular risk factors with aging. Hazard ratios with 95% confidence intervals for acute CHD or ischaemic stroke events were estimated using Cox proportional hazards model. The analysis was adjusted for study, hypertension status (≥ 140/90 mmHg or treatment), body mass index (BMI), total serum cholesterol, HDL-cholesterol and smoking status. BMI was calculated by using weight in kilograms divided by the square of height in meters and smoking status was categorized as current smoker, ex-smoker or nonsmoker. Non-diabetic women aged 40–49 years were used as the reference group for risk calculations.

## Results

The demographic data at baseline and the follow-up information in each cohort are shown in table 1. The maximum length of follow-up varied between 4.9 to 20.6 years in women and in men. During this period 182 (3.6%) women and 348 (8.4%) men had acute CHD and 129 (2.5%) women and 137 (3.3%) men acute ischaemic stroke events. A total of 384 (7.5%) women and 442 (10.6%) men had diabetes (both diagnosed and undiagnosed) (table 1). The characteristics of subjects according to age groups, diabetes status and gender are given in table 2. In the non-diabetic population, the means of BMI and total cholesterol as well as the prevalence of hypertension were all higher in men than in women in the youngest age group, but the gender differences leveled off with increasing age and became even or lower in men than in women in the oldest age group. In non-diabetic women and men the increasing trend with aging was significant (p = 0.001) for BMI, cholesterol and hypertension (table 2). Diabetic women were, however, more obese than diabetic men in all ages. HDL-cholesterol was higher in women than in men and smoking was more common in men than in women in all ages regardless of the diabetic status. The increasing trend in known CVD risk factors with aging in diabetic individuals was significant only for total cholesterol levels in women.

The event rates for acute CHD and ischaemic stroke were higher in men with and without diabetes compared with their female counterparts. The gender difference for CHD was larger in the non-diabetic than in the diabetic individuals, whereas this was not as substantial for ischaemic stroke as for CHD (table 3). The multivariate-adjusted hazard ratios of acute CHD events increased with aging and diabetes, and were higher in men both with and without diabetes compared with age matched women, but the gender difference was diminished in diabetic individuals. In contrast, the gender difference with the ischaemic stroke was enlarged in the diabetic population because the risk of ischaemic stroke increased more in diabetic men than in diabetic women (table 3). Diabetes and aging were associated with an increased risk of acute CHD and ischaemic stroke events in both genders, however diabetes was a stronger risk predictor for the risk of ischaemic stroke than for CHD (table 4). Cholesterol predicted the risk of acute CHD, but not ischaemic stroke event, in both genders. Hypertension increased the acute CHD and ischaemic stroke risk, and BMI CHD risk only in women (table 4).

## Discussion

In the present study we found that both non-diabetic and diabetic men had a higher risk of CHD than their female counterparts, but the gender difference was smaller in the diabetic than in the non-diabetic populations. In contrast, diabetic men had higher rates of ischaemic stroke events than diabetic women but the event rates did not differ much in the non-diabetic population. Diabetes conveyed a higher CHD risk in women but ischaemic stroke risk in men.

Diabetes is a well known risk factor for CHD [13-15] and stroke [16,7,8] in both genders. CHD and stroke are often discussed as diseases of the same family caused by atherosclerotic vascular disease. While this would implicate shared risk factors, studies have indicated some differences in risk factor profiles for the two diseases [9-11]. Also, the patterns of the trend of CHD and stroke incidence have been shown to vary in a population [12,17]. In the present study the diabetes attributable risk was higher to the acute ischaemic stroke than to the acute CHD in men, whereas it was similar in women. Previous studies have reported diabetes to increase the risk of CHD [2,4,5] and stroke [6-8] more markedly in women than in men. These studies have, however, used non-diabetic women and non-diabetic men as reference groups for women and for men, respectively, when they calculated the relative risk for diabetic individuals. Since the non-diabetic young men had much higher CHD and stroke risk than non-diabetic women, the relative risk increase due to diabetes in men tended to be lower than that in women. To make it comparable between women and men, we have used non-diabetic women in the youngest age group as reference for both women and men of all other age groups. We found the relative risk was higher in men than in women both with and without diabetes, even though the gender difference for the CHD risk was diminished in diabetic individuals. Contradictory to the previous reports on stroke we found in the present study a more markedly increased risk of acute ischaemic stroke events in diabetic men compared with diabetic women. But whether this is true for haemorrhagic stroke or for the subtypes of ischaemic stroke cannot be examined in the current study due to the low number of the haemorrhagic stroke events and inability of further classification of individuals into subtypes of the ischaemic stroke. Previous studies with both ischaemic and haemorrhagic stroke showed a higher relative stroke risk in diabetic women than in diabetic men [6-8], and it was also reported that the risk for certain subtypes of ischaemic or haemorrhagic stroke differed between women and men [18,19]. This might have affected the observations and needs to be explored in more detail in the future.

Non-diabetic men had higher rates of smoking, hypertension and abnormalities of lipid profiles than non-diabetic women; which could partly explain the higher CHD risk in non-diabetic men than in non-diabetic women. Also, other factors such as the menopause status [20] and the management of risk factors [20,21] may have contributed to the observed gender difference in CHD and ischemic stroke incidence. Obesity and cholesterol levels were higher in diabetic women than diabetic men, which may partly have reduced the gender difference in CHD risk in diabetic population. Aging seemed a strong risk predictor for CHD in both women and men, particularly for men. This may to some extent be explained by the fact that men are exposed to the CVD risk factors early in the life than women. Even though CHD and stroke have some common aspects, the reactivity of coronary and cerebral arteries to the CVD risk factors, environmental and genetic factors, has been found to differ [22]. Thus, as found in the present study the known CVD risk factors including diabetes may have contributed differently to the CHD and the ischaemic stroke, and partly explained the gender difference in the risk of CHD and ischaemic stroke in diabetic individuals. However, when we interpret the results we need to bear in mind that the number of ischaemic stroke events in the present study was low, particularly in the younger age groups both in women and in men.

The strengths of the present study are the long length of follow-up and the pooled data from different cohorts, which gave a large sample size and increased statistical power. In order to make up for the differences in between the different study centers all the analysis were adjusted for "cohort". Even though the sample size was relatively large, the follow-up length for many of the cohorts is still short with low numbers of the events accumulated.

## Conclusion

In summary, the CHD and ischaemic stroke risk was higher in men than in women with and without diabetes, however, the gender difference diminished for CHD but enlarged for ischaemic stroke in diabetic individuals. The known risk factors including diabetes have contributed differently to the development of CHD and ischaemic stroke in women and men. The finding may help to make strategies to manage diabetes, CHD and stroke in women and in men.

## Abbreviations

BMI: body mass index; CHD: coronary heart disease; CVD: cardiovascular disease; DECODE: Diabetes Epidemiology: Collaborative analysis Of Diagnostic criteria in Europe; FPG: fasting plasma glucose; 2-h PG: plasma glucose 2-h after 75-g glucose load

## Competing interests

The authors declare that they have no competing interests.

## Authors' contributions

MH ran the data analysis and drafted the manuscript. QQ participated in the concept, funding, design and coordination of the study and in drafting of the manuscript. TL, SS and ME took part in the data collection, drafted and approved the final version of the manuscript. PN drafted and approved the final version of the manuscript. JT was responsible for the DECODE collaboration, funding and the conception of the manuscript and approved the final version of the manuscript.

## Supplementary Material

Additional file 1 **Demographic and follow-up information of the study populations included.** Click here for file

Additional file 2 **Baseline characteristics and the incidence of cardiovascular end points in individuals according to age groups, gender, and diabetic status.** Click here for file

Additional file 3 **Event rates per 1000 person-years and hazard ratios (95% confidence intervals) for acute CHD and ischemic stroke events by diabetic status.** Click here for file

Additional file 4 **Hazard ratios (95% confidence intervals) corresponding to a one SD increase in continuous variables or as indicated.** Click here for file
