# Supplementary material for: The impact of diabetes on coronary heart disease differs from that on ischaemic stroke with regard to the gender
Source: Cardiovasc Diabetol. 2009 Mar 24;8:17. doi: 10.1186/1475-2840-8-17 (PMC2679722; doi:10.1186/1475-2840-8-17)
Supplement: Additional file 1 — Demographic and follow-up information of the study populations included. [file 1475-2840-8-17-S1.doc]

Table 1. Demographic and follow-up information of the study populations included.

| ***Study*** | ***No, (%)*** | ***Age,***  ***Median (range)*** | ***Diabetes, No (%)***  ***Prevalent Undiagnosed***  ***diabetes diabetes*** | | ***Follow-up year, (Max)*** | ***Events,No(%)***  ***CHD Ischaemic stroke*** | |
| --- | --- | --- | --- | --- | --- | --- | --- |
| ***Women*** |  |  |  |  |  |  |  |
| FINRISK-1987 | 1332 (26.1) | 53.8 (41.1-64.2) | 25 (1.9) | 44 (3.3) | 19.95 | 65 (4.9) | 101 (7.6) |
| FINRISK-1992 | 951 (18.6) | 53.9 (42.5-64.2) | 26 (2.7) | 34 (3.6) | 14.99 | 17 (1.8) | 20 (2.1) |
| FINRISK-2002 | 1745 (34.1) | 55.9 (42.0-69.0) | 69 (4.0) | 79 (4.5) | 4.94 | 6 (0.3) | 13 (0.7) |
| Vantaa | 311 (6.1) | 65.1 (64.2-66.2) | 22 (7.1) | 22 (7.1) | 13.92 | 18 (5.8) | 22 (7.1) |
| Sweden, MONICA-1986 | 189 (3.7) | 51.4 (40.0-64.0) | 11 (5.8) | 4 (2.1) | 20.61 | 10 (5.3) | 8 (4.2) |
| Sweden, MONICA-1990 | 265 (5.2) | 51.6 (40.0-64.0) | 13 (4.9) | 2 (0.8) | 16.62 | 4 (1.5) | 12 (4.5) |
| Sweden, MONICA-1994 | 318 (6.2) | 55.0 (40.0-69.0) | 13 (4.1) | 20 (6.3) | 12.62 | 9 (2.8) | 6 (1.9) |
| ***Total*** | 5111 (55.1) | 55.1 (40.0-69.0) | 179 (3.5) | 205 (4.0) | 20.61 | 129 (2.5) | 182 (3.6) |
| ***Men*** |  |  |  |  |  |  |  |
| FINRISK-1987 | 1093 (26.2) | 53.5 (43.6-64.2) | 32 (2.9) | 25 (2.3) | 19.95 | 64 (5.9) | 169 (15.5) |
| FINRISK-1992 | 745 (17.9) | 53.5 (41.2-64.1) | 20 (2.7) | 42 (5.6) | 14.95 | 20 (2.7) | 52 (7.0) |
| FINRISK-2002 | 1378 (33.1) | 56.6 (41.0-69.0) | 81 (5.9) | 136 (9.9) | 4.94 | 9 (0.7) | 26 (1.9) |
| Vantaa | 221 (5.3) | 65.1 (64.2-66.1) | 19 (8.6) | 8 (3.6) | 13.94 | 24 (10.9) | 32 (14.5) |
| Sweden, MONICA-1986 | 189 (4.5) | 51.8 (40.0-64.0) | 22 (11.6) | 4 (2.1) | 20.61 | 5 (2.6) | 22 (11.6) |
| Sweden, MONICA-1990 | 245 (5.9) | 51.8 (40.0-64.0) | 20 (8.2) | 3 (1.2) | 16.62 | 9 (3.7) | 20 (8.2) |
| Sweden, MONICA-1994 | 296 (7.1) | 54.8 (40.0-69.0) | 14 (4.7) | 16 (5.4) | 12.62 | 6 (2.0) | 27 (9.1) |
| ***Total*** | 4167 (44.9) | 55.1 (40.0-69.0) | 208 (5.0) | 234 (5.6) | 20.61 | 137 (3.3) | 348 (8.4) |
