# Supplementary material for: The impact of diabetes on coronary heart disease differs from that on ischaemic stroke with regard to the gender
Source: Cardiovasc Diabetol. 2009 Mar 24;8:17. doi: 10.1186/1475-2840-8-17 (PMC2679722; doi:10.1186/1475-2840-8-17)
Supplement: Additional file 2 — Baseline characteristics and the incidence of cardiovascular end points in individuals according to age groups, gender, and diabetic status. [file 1475-2840-8-17-S2.doc]

|  | ***Age Groups, years*** | | | | | | | | |
| --- | --- | --- | --- | --- | --- | --- | --- | --- | --- |
|  | **40-49** | |  | **50-59** | |  | **60-69** | |  |
| **Non-diabetic** | **Women** | **Men** | ***p-value*** | **Women** | **Men** | ***p-value*** | **Women** | **Men** | ***p-value*** |
| No, (%) | 1316 (95.4) | 1060 (94.2) |  | 2080 (93.4) | 1642 (89.4) |  | 1331 (88.5) | 1023 (84.9) |  |
| BMI (Kg/m^2^) | 25.87 (0.12) | 26.75 (0.11) | *<0.001* | 27.28 (0.10) | 27.45 (0.09) | *0.226* | 27.78 (0.13) | 27.11 (0.11) | *<0.001* |
| Cholesterol (mmol/L) | 5.69 (0.03) | 6.18 (0.03) | *<0.001* | 6.23 (0.03) | 6.18 (0.03) | *0.072* | 6.44 (0.03) | 6.02 (0.03) | *<0.001* |
| HDL (mmol/L) | 1.57 (0.01) | 1.29 (0.01) | *<0.001* | 1.62 (0.01) | 1.31 (0.01) | *<0.001* | 1.57 (0.01) | 1.28 (0.01) | *<0.001* |
| Hypertension (Yes/No) | 537 (40.8) | 551 (52.0) | *<0.001* | 1270 (61.1) | 1085 (66.1) | *0.002* | 988 (74.2) | 766 (74.9) | *0.721* |
| Smoking vs. non-smoking | 316 (24.0) | 322 (30.4) | *<0.001* | 374 (18.0) | 488 (29.7) | *<0.001* | 177 (13.3) | 240 (23.5) | *<0.001* |
| CHD | 20 (1.5) | 47 (4.4) | *<0.001* | 57 (2.7) | 132 (8.0) | *<0.001* | 68 (5.1) | 114 (11.1) | *<0.001* |
| Ischaemic stroke | 12 (0.9) | 16 (1.5) | *0.180* | 43 (2.1) | 48 (2.9) | *0.093* | 52 (3.9) | 44 (4.3) | *0.632* |
| **Diabetic** |  |  |  |  |  |  |  |  |  |
| No, (%) | 64 (4.6) | 65 (5.8) |  | 147 (6.6) | 195 (10.6) |  | 173 (11.5) | 182 (15.1) |  |
| BMI (Kg/m^2^) | 30.51 (0.83) | 29.85 (0.65) | *0.584* | 31.86 (0.48) | 29.74 (0.31) | *<0.001* | 30.41 (0.39) | 29.25 (0.31) | *0.017* |
| Cholesterol (mmol/L) | 5.88 (0.12) | 6.24 (0.15) | *0.077* | 6.11 (0.09) | 5.94 (0.10) | *0.417* | 6.43 (0.09) | 5.95 (0.09) | *0.007* |
| HDL (mmol/L) | 1.46 (0.06) | 1.20 (0.05) | *<0.001* | 1.41 (0.03) | 1.24 (0.03) | *<0.001* | 1.34 (0.03) | 1.23 (0.02) | *<0.001* |
| Hypertension (Yes/No) | 38 (59.4) | 49 (75.4) | *0.052* | 117 (79.6) | 162 (83.1) | *0.410* | 150 (86.7) | 154 (84.6) | *0.575* |
| Smoking vs. non-smoking | 20 (31.3) | 28 (43.1) | *0.117* | 32 (21.8) | 62 (31.8) | *<0.001* | 23 (13.3) | 40 (22.0) | *<0.001* |
| CHD | 4 (6.3) | 7 (10.8) | *0.358* | 12 (8.2) | 23 (11.8) | *0.273* | 21 (12.1) | 25 (13.7) | *0.654* |
| Ischaemic stroke | 2 (3.1) | 3 (4.6) | *0.661* | 4 (2.7) | 9 (4.6) | *0.364* | 16 (9.2) | 17 (9.3) | *0.976* |

Table 2. Baseline characteristics and the incidence of cardiovascular end points in individuals according to age groups, gender, and diabetic status.

Data are given as means (standard error) adjusted for age, study and sex, or as number (%).

p-values reflect differences between men and women.
