# Supplementary material for: The impact of diabetes on coronary heart disease differs from that on ischaemic stroke with regard to the gender
Source: Cardiovasc Diabetol. 2009 Mar 24;8:17. doi: 10.1186/1475-2840-8-17 (PMC2679722; doi:10.1186/1475-2840-8-17)
Supplement: Additional file 3 — Event rates per 1000 person-years and hazard ratios (95% confidence intervals) for acute CHD and ischemic stroke events by diabetic status. [file 1475-2840-8-17-S3.doc]

Table 3. Event rates per 1000 person-years and hazard ratios (95% confidence intervals) for acute CHD and ischaemic stroke events by diabetic status.

|  | ***Non-diabetic*** | | | ***Diabetic*** | | |
| --- | --- | --- | --- | --- | --- | --- |
|  |  | **Age groups, yr** |  |  | **Age groups, yr** |  |
|  | **40-49** | **50-59** | **60-69** | **40-49** | **50-59** | **60-69** |
| **CHD** |  |  |  |  |  |  |
| **Rates** |  |  |  |  |  |  |
| Women | 1.09 (0.68-1.65) | 2.20 (1.68-2.83) | 4.61 (3.61-5.81) | 5.68 (1.80-13.69) | 7.50 (4.06-12.74) | 12.53 (7.96-18.82) |
| Men | 3.11 (2.31-4.10) | 6.57 (5.52-7.77) | 10.82 (8.97-12.95) | 9.62 (4.21-19.03) | 13.24 (8.59-19.55) | 17.51 (11.59-25.47) |
| **HRs** |  |  |  |  |  |  |
| **Model 1** |  |  |  |  |  |  |
| Women | 1 | 2.10 (1.26-3.49) | 4.91 (2.97-8.12) | 6.09 (2.08-17.82) | 7.95 (3.88-16.26) | 14.65 (7.87-27.28) |
| Men | 2.90 (1.72-4.90) | 6.43 (4.02-10.29) | 12.34 (7.62-19.97) | 10.44 (4.41-24.70) | 15.98 (8.76-29.15) | 22.54 (12.41-40.97) |
| **Model 2** |  |  |  |  |  |  |
| Women | 1 | 1.78 (1.06-2.97) | 3.75 (2.24-6.26) | 4.35 (1.48-12.80) | 5.49 (2.66-11.33) | 8.84 (4.68-16.72) |
| Men | 1.94 (1.14-3.29) | 4.23 (2.61-6.84) | 8.40 (5.13-13.76) | 5.40 (2.26-12.93) | 9.54 (5.17-17.63) | 13.76 (7.47-25.34) |
| **Ischaemic stroke** |  |  |  |  |  |  |
| **Rates** |  |  |  |  |  |  |
| Women | 0.65 (0.35-1.11) | 1.66 (1.22-2.22) | 3.53 (2.66-4.59) | 2.84 (0.48-9.38) | 2.50 (0.79-6.03) | 9.54 (5.65-15.17) |
| Men | 1.06 (0.63-1.68) | 2.39 (1.78-3.14) | 4.18 (3.07-5.56) | 4.12 (1.05-11.22) | 5.18 (2.53-9.50) | 11.91 (7.17-18.68) |
| **HRs** |  |  |  |  |  |  |
| **Model 1** |  |  |  |  |  |  |
| Women | 1 | 2.62 (1.38-4.97) | 5.71 (3.01-10.81) | 5.08 (1.14-22.72) | 4.23 (1.36-13.12) | 17.78 (8.26-38.30) |
| Men | 1.63 (0.77-3.43) | 3.86 (2.05-7.26) | 7.15 (3.73-13.69) | 7.87 (2.22-27.93) | 10.60 (4.46-25.20) | 27.22 (12.76-58.10) |
| **Model 2** |  |  |  |  |  |  |
| Women | 1 | 2.48 (1.30-4.73) | 5.17 (2.69-9.94) | 4.14 (0.92-18.66) | 3.32 (1.06-10.43) | 13.91 (6.31-30.66) |
| Men | 1.26 (0.59-2.70) | 2.83 (1.48-5.42) | 5.11 (2.62-9.97) | 4.91 (1.36-17.74) | 6.75 (2.79-16.32) | 18.06 (8.29-39.37) |

**Model 1** Adjusted for study

**Model 2** Adjusted for study, BMI, hypertension, total cholesterol, HDL-cholesterol, smoking
