# Supplementary material for: The impact of diabetes on coronary heart disease differs from that on ischaemic stroke with regard to the gender
Source: Cardiovasc Diabetol. 2009 Mar 24;8:17. doi: 10.1186/1475-2840-8-17 (PMC2679722; doi:10.1186/1475-2840-8-17)
Supplement: Additional file 4 — Hazard ratios (95% confidence intervals) corresponding to a one SD increase in continuous variables or as indicated. [file 1475-2840-8-17-S4.doc]

Table 4. Hazard ratios (95% confidence intervals) corresponding to a one SD increase in continuous variables or as indicated.

|  | **CHD** | | **Ischaemic stroke** | |
| --- | --- | --- | --- | --- |
|  | **Women** | **Men** | **Women** | **Men** |
| Age, 40-49 yrs | 1 | 1 | 1 | 1 |
| Age, 50-59 yrs | 1.70 (1.06-2.75) | 2.16 (1.58-2.95) | 2.16 (1.17-3.98) | 2.12 (1.26-3.58) |
| Age, 60-69 yrs | 3.53 (2.15-5.79) | 3.99 (2.85-5.58) | 5.49 (2.93-10.28) | 3.63 (2.06-6.38) |
| BMI, kg/m^2^ | 1.22 (1.06-1.40) | 1.02 (0.91-1.14) | 0.97 (0.81-1.16) | 1.05 (0.88-1.26) |
| Cholesterol, mmol/l | 1.25 (1.09-1.43) | 1.34 (1.22-1.49) | 0.94 (0.79-1.12) | 1.00 (0.85-1.19) |
| HDL, mmol/l | 0.71 (0.59-0.85) | 0.84 (0.74-0.94) | 0.79 (0.64-0.97) | 0.90 (0.75-1.09) |
| Hypertension (Yes/No) | 1.51 (1.03-2.21) | 1.27 (0.97-1.65) | 2.33 (1.42-3.81) | 1.47 (0.94-2.30) |
| Smoking vs. non-smoking | 2.81 (1.97-4.01) | 1.95 (1.51-2.52) | 1.16 (0.67-1.99) | 2.06 (1.33-3.19) |
| Diabetes mellitus (Yes/No) | 2.48 (1.69-3.65) | 2.09 (1.55-2.82) | 2.37 (1.46-3.84) | 3.01 (1.95-4.64) |
